# Risk of autism spectrum disorder in children with infantile epileptic spasms syndrome: a retrospective study in a single center in Brazil

**DOI:** 10.1016/j.jped.2024.04.006

**Published:** 2024-05-29

**Authors:** Marília Barbosa de Matos, Paulo Breno Noronha Liberalesso, Tiago dos Santos Bara, Paula Carolina Martins Alves Gomes, Bianca Simone Zeigelboim, Jair Mendes Marques, Mara L. Cordeiro

**Affiliations:** a Instituto de Pesquisa Pelé Pequeno Príncipe, Curitiba, PR, Brazil; b Faculdades Pequeno Príncipe, Curitiba, PR, Brazil; c Hospital Pequeno Príncipe, Departamento de Neurologia Pediátrica, Curitiba, PR, Brazil; d Universidade Tuiuti do Paraná, Laboratório de Otoneurologia, Curitiba, PR, Brazil; e Centro de Reabilitação Neuropediátrica do Hospital Menino Deus (CERENA), Curitiba, PR, Brazil

**Keywords:** Autism spectrum disorder, Infantile epileptic spasms syndrome, Hypsarrhythmia, Seizures

## Abstract

**Objective:**

This study aimed to investigate the prevalence of autism spectrum disorder and its possible correlations with clinical characteristics in patients with infantile epileptic spasms syndrome in a single center in Brazil.

**Methods:**

This retrospective cross-sectional study examined 53 children with the diagnosis of infantile epileptic spasms syndrome prior to an autism spectrum disorder assessment. Participants were divided into two groups based on the presence or absence of autism spectrum disorder. Available variables (sex, medications, median age at onset of infantile epileptic spasms syndrome, and presence of comorbidities) were compared using Mann–Whitney U or chi-square tests.

**Results:**

Among the included patients, 12 (23 %) were diagnosed with autism spectrum disorder, corresponding to a relative risk of 0.29 (95 % confidence interval 0.174–0.492). The age at the first seizure ranged from 3 to 15 months, with a mean of 6.65 months. This age significantly differed between participants with autism spectrum disorder (10.58 months) and those without (5.43 months), *p*<0.001.

**Conclusion:**

Children with infantile epileptic spasms syndrome have a higher risk of being diagnosed with autism spectrum disorder. Later age of onset and period of spasm occurrence might be predisposing risk factors.

## Introduction

The correlation between epilepsy and autism spectrum disorder (ASD) has already been established,^1^ and several studies have reported an ASD diagnosis in 25.0–46.2 % of patients with infantile epileptic spasms syndrome (IESS).^2^, ^3^, ^4^, ^5^

The new ILEA classification proposes the use of the term IESS to encompass West syndrome and epileptic spasms that do not fulfill all criteria of a West syndrome. West syndrome is defined as the presence of epileptic spasms, hypsarrhythmia and developmental stagnation or regression. It starts between 1 and 24 months of age and peaks at approximately 3–12 months of age. Before IESS onset, development can be normal; however, a history of abnormal development is often found. This is the most frequent form of epileptic encephalopathy in the first year of life, with an estimated incidence of 30/100,000 live-born infants.^6^

ASD is a complex neurodevelopment condition in which genetic and environmental factors contribute to its etiology.^7^ The prevalence of ASD among infants with IESS exceeds the prevalence of ASD in the general population, which is approximately 1:36 according to the Centers for Disease Control and Prevention (CDC).^8^

Emerging evidence suggests a neuropathological link between IESS and ASD, particularly in patients with a known genetic cause; genetic abnormalities might play a role in the child's neurodevelopment outcomes. Furthermore, epileptic encephalopathy, a potential consequence of IESS, disrupts brain development through alterations in neurotransmitter systems and abnormal electrical activity, potentially contributing to ASD in some children.^9^ Common comorbidities of IESS like tuberous sclerosis or cerebral palsy might be important for understanding ASD etiology in these patients.^1^^,^^10^ Although previous studies have reported an association between IESS and ASD,^2^, ^3^, ^4^, ^5^ data from developing countries such as Brazil are lacking.

Therefore, this study aimed to determine the prevalence of ASD among children with IESS in a single center in Brazil. Additionally, the authors aimed to delineate the characteristics of children with IESS and the potential mediators underlying the correlation between IESS and ASD.

## Methods

### Study design and study population

This retrospective cross-sectional study based on the assessment of medical records from the Department of Pediatric Neurology of the Pequeno Príncipe Hospital, Curitiba, PR, Brasil between January 2007 and January 2017 focused on a single time point. All research was approved by the Ethics Committee on Research Involving Human Subjects of the present study's institution (CAAE 80683917.7.0000.0097). Written informed consent was obtained from the patient's parents for the publication of this report.

This study included patients with a history of regular attendance at an outpatient neuropediatric clinic and a conclusive diagnosis of IESS based on the presence of infantile spasms, neurodevelopmental delay, and at least one electroencephalogram (EEG) with hypsarrhythmia. The study population was divided into two groups—patients with and without ASD—to compare their demographic and clinical data.

### Data collection and outcome measures

The authors evaluated the medical records of all patients with a history of regular attendance at outpatient neuropediatric clinics (usually every 3 months, with an average of four visits per year) for extraction of the following clinical variables: sex, age at IESS diagnosis, medications, presence of comorbidities and complementary diagnostic test results (EEG, brainstem auditory evoked potential and neuroimaging data).

Data were extracted from the first EEG that was consistent with hypsarrhythmia for a formal diagnosis of IESS and from the last EEG of the evaluation period, which was arbitrarily defined as 2 years. EEGs were performed on all patients at outpatient neuropediatric clinics for a minimum duration of 40 min, with electrodes positioned according to the International 10–20 System. All EEGs were analyzed by the same neurophysiologist. Hypsarrhythmia was defined as chaotic, high amplitude, excessively slowing, multifocal epileptiform discharges that typically were attenuated or stopped during a series of epileptic spasms.^6^

The assessment of ASD was performed when clinical suspicion arose during the evaluation sessions and was based on detailed clinical history **(**including information about the child's development, medical history, family history and current behaviors) provided by parents, caregivers, or other relevant individuals. In addition, the child's communication and interaction skills were directly observed during semi-structured play. Supplemental information was based on the Childhood Autism Rating Scale (CARS).^11^ The final diagnosis was based on the DSM-5 criteria^12^ and was made by a senior child neurologist, with the support of a clinical neuropsychologist.

### Statistical analysis

Data were compiled in a Microsoft Excel 2013 spreadsheet and analyzed using SPSS for Windows version 23. Measures of central tendency were calculated for all variables. The relative risk (RR) was calculated to assess the association between patients with and without ASD. To compare groups with and without ASD, Mann–Whitney U tests for continuous variables and chi-square tests for categorical variables were employed. A significance level of alpha (α) < 0.05 was used for all analyses.

## Results

Of the 53 participants diagnosed with IESS, 29 (55 %) were female, and 24 (45 %) were male. The mean age at the time of data collection was 49.06 ± 10.06 months, ranging from 28 to 72 months. Regarding comorbidities, five (9.4 %) participants had tuberous sclerosis, seven (13.2 %) had Down's syndrome, 15 (28.3 %) had cerebral palsy, 13 (24.5 %) had cortical malformation, 10 (18.7 %) had mild magnetic resonance imaging abnormalities due to prematurity and three (5.6 %) were classified as having idiopathic IESS.

The age at the first seizure ranged from 3 to 15 months (mean, 6.65 ± 2.71 months), and treatment was initiated after the diagnosis of IESS. The first-line medications included vigabatrin (86.6 %) and adrenocorticotropic hormone (ACTH; 13.2 %), whereas a second-line drug was either added or substituted in 38 patients (71.7 %). Persistence of spasms or evolution to other types of seizures one year after diagnosis was observed in 24 patients. Moreover, hypsarrhythmia on EEG persisted two years after diagnosis in 16 patients.

In the study population, 12 (23 %) of the 53 participants were diagnosed with ASD. Table 1 shows the clinical and demographic data of the participants with ASD. The RR of an ASD diagnosis in children with IESS was 0.293 (95 % CI 0.174–0.492).

**Table 1:** Characteristics of the study participants with ASD.

	Sex	Age (months)	Age at first spasm (months)	MRI	Comorbidities	First-line drug
**1**	Male	39	9	Corpus callosum agenesia	Cerebral malformation	Vigabatrin
**2**	Female	51	8	Multicysticencephalomalacia	HII	Vigabatrin
**3**	Male	47	11	Cerebral atrophy	HII	ACTH
**4**	Female	63	15	Cerebral atrophy	HII	Vigabatrin
**5**	Female	49	9	Normal	Down's syndrome	Vigabatrin
**6**	Male	60	9	Periventricular leucomalacia	Prematurity	Vigabatrin
**7**	Female	51	11	Periventricular leucomalacia	Prematurity	ACTH
**8**	Male	50	13	Cerebral atrophy	HII	Vigabatrin
**9**	Male	38	10	Periventricular leukomalacia	Prematurity	Vigabatrin
**10**	Male	57	11	Normal	Down's syndrome	Vigabatrin
**11**	Male	58	9	Normal	Down's syndrome	ACTH
**12**	Male	70	12	Periventricular tubers	Tuberous sclerosis	Vigabatrin

ACTH, adrenocorticotropic hormone; ASD, autism spectrum disorder; HII, hypoxic-ischemic brain injury; Cryomagnetic resonance imaging.

The study population was divided into two groups: those with ASD (Group 1) and those without ASD (Group 2). A comparison of the clinical variables is presented in Table 2. None of the children with idiopathic IESS were subsequently diagnosed with ASD.

**Table 2:** Comparison of children with and without ASD.

Variables (%)	With ASD (*n* = 12)	Without ASD (*n* = 41)	P
Sex, male	58.33	41.46	0.306
Prematurity	25.00	17.07	0.537
Cerebral palsy	58.33	43.90	0.378
Cerebral malformation	8.33	29.26	0.142
Syndrome*	33.33	19.51	0.314
Seizure control	41.66	58.53	0.301
Hypsarrhythmia	33.33	29.26	0.787
More than one drug used	91.66	65.85	0.805

⁎ Including Down's syndrome and tuberous sclerosis. ASD, autism spectrum disorder.

The median (minimum-maximum range) age of spasm onset was significantly different between the two groups: 10.58 (8–15) months in Group 1 and 5.43 (3–8) months in Group 2 (*p* < 0.001). None of the participants with spasm onset before the age of six months had an ASD diagnosis.

## Discussion

This retrospective cross-sectional study showed that, of the 53 enrolled patients with IESS, 12 (23 %) were diagnosed with ASD, resulting in an RR of 0.29 (95 % CI 0.174–0.492). These findings are consistent with the results of similar studies from France and Iceland [2–5] and show a substantially higher ASD prevalence compared with the latest prevalence rate in the general population reported by the CDC.^8^ To the best of our knowledge, the current study is the first to report the prevalence of ASD among children with IESS in South America.

Another interesting finding of this study was that children with later-onset spasms (approximately at 10 months of age) were more likely to receive an ASD diagnosis, whereas none of the children diagnosed with IESS before 6 months of age were diagnosed with ASD. This is an unexpected result since prior studies typically reported greater neurodevelopmental deterioration in patients with earlier-onset spasms.^12^^,^^13^

Previous studies have suggested that epileptic seizures can trigger maladaptive synaptic plasticity, leading to imbalances between excitatory and inhibitory neurotransmitter systems, thereby contributing to neurodevelopmental impairments.^14^ The pruning process occurs primarily during the first year of life, with sensory and motor areas particularly vulnerable between ages 6 and 12 months,^15^ and abnormalities in these processes have already been associated with ASD diagnosis.^16^ The authors therefore hypothesize that the emergence of severe electrophysiological dysfunction, as hypsarrhythmia, involving cortical and subcortical regions during this critical period may substantially increase the risk of ASD development in children with IESS.

Another possible explanation for our findings is the fact that the age of onset is based on the date of referral by the parents. Since infantile spasms may appear as subtle movements, they can be easily missed or misinterpreted as a colic or irritation leading to a later diagnosis and treatment.^17^^,^^18^ This delay might be particularly critical as the persistence of spasms beyond 90 days has been linked to poorer developmental outcomes.^19^

In the present study, the persistence of spasms or hypsarrhythmia and the evolution to another type of seizure in the second year of life did not lead to a subsequent ASD diagnosis. In contrast, another study in children with IESS showed significant persistent hypsarrhythimia in children with ASD at or after one year of age.^20^

This study also explored potential links to pre-existing structural brain abnormalities or the presence of genetic disorders, known risk factors for ASD in patients with IESS.^10^^,^^21^, ^22^, ^23^ While the presence of these comorbidities did not significantly differ between children with and without ASD, it is noteworthy that none of the children diagnosed with idiopathic IESS developed ASD later. A recent systematic review of autism and epilepsy demonstrated that individuals with secondary epilepsy have a higher risk of ASD than those with idiopathic epilepsy, and this association may be the result of a mutual underlying neurological condition.^1^

Regarding the treatment, no association was found between the choice of first-line medication or the use of additional medications and the development of ASD. This aligns with previous findings from a randomized controlled trial, which showed that early and effective treatment of IESS does not necessarily reduce the risk of ASD.^24^ However, a study in Iran reported that over half of the children with IESS experienced significant developmental delays, particularly those with persistent seizures or symptomatic IESS.^22^

The present study has some limitations. Although the sample size might be sufficient for exploratory studies, it poses several challenges for drawing valid conclusions about a broader population due to reduced statistical power, limited generalizability, and the possibility of an increased sampling error. The generalizability of these findings is limited by two factors. First, the symptom severity of the enrolled participants may not be representative of the entire IESS population. Second, all participants had intellectual disability as a comorbid condition. Finally, the authors acknowledge that the gold-standard diagnostic instruments, ADOS-2 and ADI-R, were not employed due to the lack of appropriate validation for the Brazilian population.^25^, ^26^, ^27^ Nonetheless, this study has some strengths. First, it represents the largest sample size investigated to date regarding ASD prevalence in children with IESS. Second, all patients were examined in a neurological pediatric center by specialists in epileptology and neurodevelopmental disorders.

In conclusion, the present data contribute to the sparse knowledge regarding the link between IESS and ASD. The authors found a higher prevalence of ASD among children with IESS compared to that among the general population. Notably, these findings suggest a potential link between a later age of spasm onset (approximately 10.58 months) and the risk of an ASD diagnosis. Neurodevelopment is influenced by multiple factors, and future research with larger, more generalizable cohorts is needed to confirm the present study's findings, identify risk factors, and better elucidate the underlying mechanisms.

The authors suggest that after the diagnosis of IESS, these children should be screened and assessed for ASD to enable earlier detection and a better prognosis. The nature of this association, whether spasms contribute to its etiology or whether a common genetic basis is present, is not yet completely understood. Further longitudinal studies with a larger population and inclusion of control groups should be performed to better understand this important association.

## Conflicts of interest

The authors declare no conflicts of interest.
